# Overall and cause-specific excess mortality in HIV-positive persons compared with the general population: Role of HCV coinfection

**DOI:** 10.1097/MD.0000000000004727

**Published:** 2016-09-09

**Authors:** Belén Alejos, Victoria Hernando, Jose Iribarren, Juan Gonzalez-García, Asuncion Hernando, Jesus Santos, Victor Asensi, Ana Gomez-Berrocal, Julia del Amo, Inma Jarrin, CoRIS (Cohort of the Spanish Network on HIV/AIDS Research)

**Affiliations:** a National Center of Epidemiology, Instituto de Salud Carlos III; b Hospital Universitario de Donostia, Donostia; c Hospital La Paz; d Hospital, Doce Octubre, Madrid; e Hospital, Virgen de la Victoria, Malaga; f Hospital, Central de Asturias, Oviedo; g Hospital La Princesa, Madrid, Spain.

**Keywords:** antiretroviral therapy, cause of death, cohort studies, hepatitis C, highly active, HIV

## Abstract

We aimed to estimate overall and cause-specific excess mortality of HIV-positive patients compared with the general population, and to assess the effect of risk factors.

We included patients aged >19 years, recruited from January 1, 2004 to May 31, 2014 in Cohort of the Spanish Network on HIV/AIDS Research. We used generalized linear models with Poisson error structure to model excess mortality rates.

In 10,340 patients, 368 deaths occurred. Excess mortality was 0.82 deaths per 100 person-years for all-cause mortality, 0.11 for liver, 0.08 for non-AIDS-defining malignancies (NADMs), 0.08 for non-AIDS infections, and 0.02 for cardiovascular-related causes. Lower CD4 count and higher HIV viral load, lower education, being male, and over 50 years were predictors of overall excess mortality. Short-term (first year follow-up) overall excess hazard ratio (eHR) for subjects with AIDS at entry was 3.71 (95% confidence interval [CI] 2.66, 5.19) and 1.37 (95% CI 0.87, 2.15) for hepatitis C virus (HCV)-coinfected; medium/long-term eHR for AIDS at entry was 0.90 (95% CI 0.58, 1.39) and 3.83 (95% CI 2.37, 6.19) for HCV coinfection. Liver excess mortality was associated with low CD4 counts and HCV coinfection. Patients aged ≥50 years and HCV-coinfected showed higher NADM excess mortality, and HCV-coinfected patients showed increased non-AIDS infections excess mortality.

Overall, liver, NADM, non-AIDS infections, and cardiovascular excesses of mortality associated with being HIV-positive were found, and HCV coinfection and immunodeficiency played significant roles. Differential short and medium/long-term effects of AIDS at entry and HCV coinfection were found for overall excess mortality.

## Abstract 2

Supplemental Digital Content is available in the text

## 1 Introduction

Since the introduction of combination antiretroviral therapy (cART), the life expectancy of HIV-positive individuals starting cART and attaining restoration of CD4 approaches that of the general population.^[1]^ However, excess mortality, that is, the mortality above what would be expected in the general population due to both AIDS and non-AIDS-defining conditions, remains as duration of HIV infection lengthens.^[2–4]^ Hepatitis C virus (HCV) and/or hepatitis B virus (HBV) coinfection and tobacco use, together with long exposure to cART and long-standing HIV replication, may further contribute to the diversification of morbidity and mortality.^[5–7]^ Studies from several European countries have reported an important excess mortality in HIV/HCV-coinfected patients.^[8,9]^ However, it is essential to determine the contribution of HCV coinfection to an increased cause-specific mortality in HIV-positive patients. Whereas most available data on excess mortality refer to all-cause mortality, less data are available on the cause-specific excess mortality associated with being HIV-positive. This is important given that non-AIDS-defining malignancies (NADMs), cardiovascular disease, and liver-related deaths have become more frequent as a consequence of more prolonged survival of an increasingly HIV-positive population.^[2,10]^

Therefore, we aimed to evaluate the overall and cause-specific excess mortality observed in HIV-positive subjects followed up in the cohort of the Spanish Network on HIV/AIDS Research (CoRIS) from 2004 to 2014, compared with the expected mortality in the general population in Spain, and to identify prognostic factors of excess mortality.

## 2 Methods

### 2.1 Subjects

The CoRIS cohort or CoRIS is an open, multicentre, prospective cohort of HIV-positive adults recruited while cART-naïve from 40 centers from 13 of the 17 Autonomous Communities of Spain.^[11]^ Ethics approval was obtained from all hospitals Ethics Committees, and every patient provided written informed consent to participate in the cohort.

For this analysis, we included patients older than 19 years recruited from January 1, 2004 to May 31, 2014 (administrative censoring date). Individuals were followed up from study entry to death or last study contact, whichever arose first.

### 2.2 Definition of variables

We considered the following variables: age at entry (20–49, ≥50); sex (male, female); HIV transmission category (injection drug users [IDUs], men who have sex with men [MSM], heterosexual contact, others); level of education (no education or compulsory [which included primary and lower secondary], upper secondary, university, others [no possible to classify according this system]); geographical region of origin (Spain, Latin America, sub-Saharan Africa, others); AIDS-defining condition at entry, CD4 cells/mm^3^ count at entry (<200, 200–350, >350); HIV viral load (VL) at entry copies/mL (<100000, ≥100000); HCV serological status at entry (positive or negative antibodies); cART initiation in the first 6 months after cohort entry; vital status; cause and date of death. Follow-up time was divided in 2 intervals: first year after the inclusion in the cohort to account for short-term mortality, and from the second year to the 10th year to account for medium/long-term mortality.

### 2.3 Classification of deaths in the general population and in the cohort

Mortality rates in the general population, from 2004 to 2013, were obtained from the National Institute of Statistics (www.ine.es), stratified by sex and age at 5-year intervals. A constant death rate within each 5-year age interval was assumed.

Deaths were classified using revised CoDe in CoRIS and 10th revision of the International Classification of Diseases (ICD-10) was applied in the general population and grouped as: liver disease (including HCV and HBV-related liver cancers), NADM, non-AIDS infections, and cardiovascular disease (see Table 1).^[12]^ Revised CoDe is a simplified version of CoDe coding system that has been proposed by the Antiretroviral Therapy Cohort Collaboration (ART-CC),^[13]^ which has been previously applied to CoRIS.^[12]^

**Table 1:**
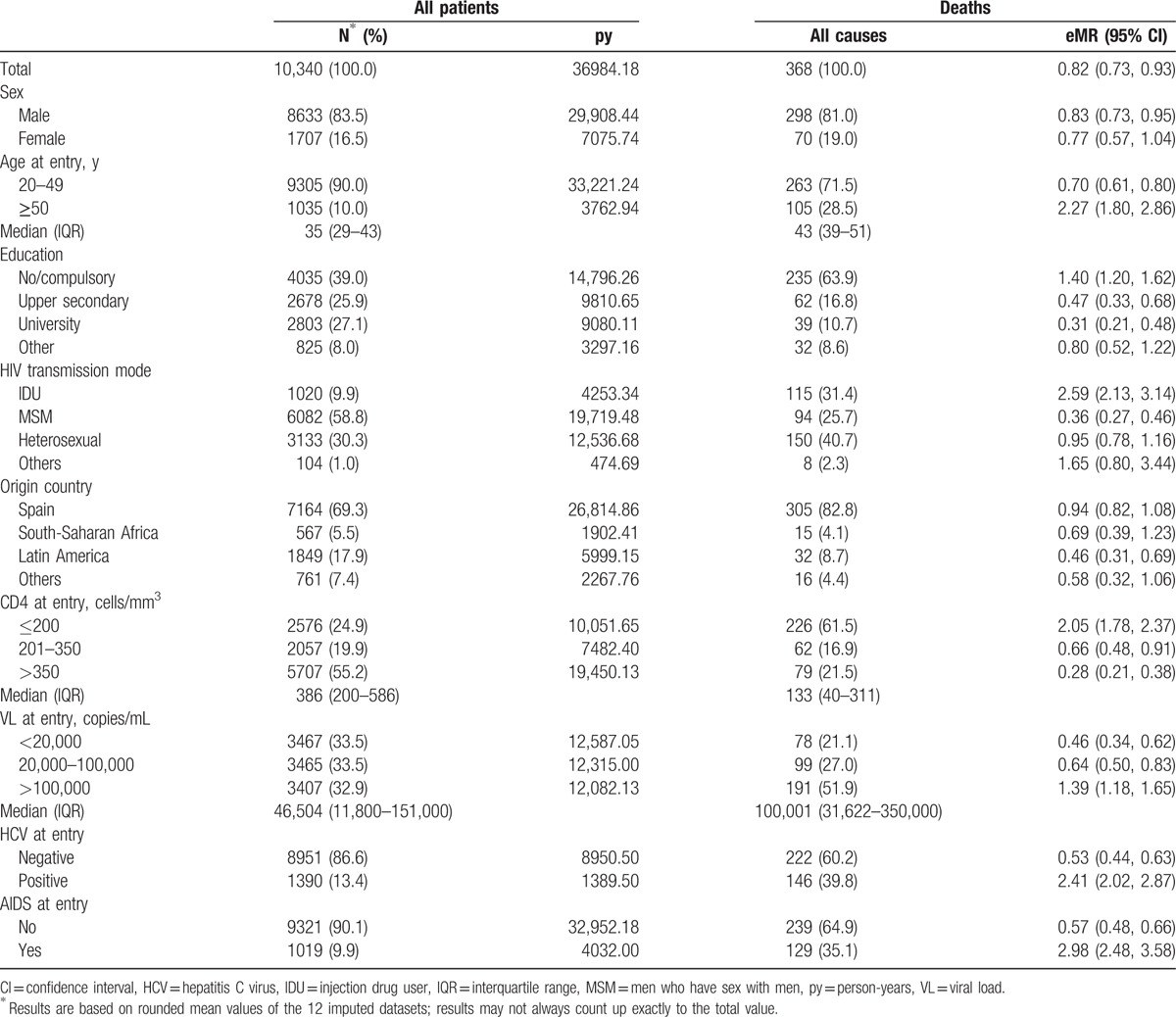
Sociodemographic and clinical characteristics of patients included by vital status and overall excess mortality rates (eMRs) per 100 person-years of follow-up.

### 2.4 Statistical analysis

Descriptive analysis of patients characteristics was performed using frequency distributions for categorical variables and median (interquartile range [IQR]) for continuous variables for all and deceased patients.

Excess mortality rate associated with being HIV-positive is defined as the difference between the death rate observed in the cohort and the expected death rate in the general population. The expected number of deaths was calculated by applying the mortality rates of the general population to the person-years (py) distribution of the HIV cohort matched by age, sex, and calendar year at risk. We used multivariable generalized linear models with Poisson error structure to estimate both the excess mortality rates (eMRs) in HIV-positive patients compared with the general population and the excess hazard ratio (eHR) for potential prognostic factors. This latter estimation should be interpreted as a common HR, but in terms of excess mortality.

A competing risk analog of the generalized linear model with Poisson error structure was applied to estimate cause-specific excess mortality in HIV-positive patients. We fitted separated models for liver, NADM, non-AIDS infections, and cardiovascular deaths; individuals who developed a competing event before the event of interest were censored on the occurrence date of the competing event.

In the multivariable models, we included all the prognostic factors with *P* values <0.1 in the crude analysis, CD4 count at entry, and follow-up interval (to assume piecewise constant hazards in each interval). We checked the sensibility of these assumption using 1-year intervals of follow-up (see Supplementary Fig. 1). Further, to investigate whether the effects of the prognostic factors on overall and cause-specific excess mortality differed by follow-up interval, interaction terms between follow-up intervals and prognostic factors were tested. Due to the small number of events and to avoid overfitting, we did not consider the variable “AIDS at entry” for NADM, liver, and non-AIDS infections multivariable models. We also checked that the number of observed deaths was above the expected deaths in each subgroup.

“Multiple Imputation by Chained Equations” was used to deal with missing data. We developed a multiple imputation model for each variable with missing values. Resulting models included the other incomplete variables (education, HIV transmission, origin region, CD4, VL, HCV, cause of death); the complete variables (AIDS at entry, age, sex); the outcome (survival time, cause of death); and 1 additional variable “receiving cART in the first 6 months after cohort entry). The results from the 12 imputed datasets were combined using Rubin rules.^[14]^ We assessed the imputation procedure and its convergence. Finally, we performed exhaustive sensitivity analysis comparing results obtained using imputed values and using only complete cases or excluding individuals with imputed outcomes.

All statistical analyses were performed using STATA (Version 13.0, College Station, TX).

## 3 Results

### 3.1 Characteristics of the participants

The total number of patients older than 19 years included in CoRIS from January 1, 2004 to May 31, 2014 was 10,340. The median follow-up was 3.20 years (IQR 1.01, 5.66), and during 36,984.18 py of follow-up, 368 deaths were observed. The distribution of causes of death in CoRIS and the general population is presented in Supplementary Table 1.

We found missing data on level of education (1349, 13.1%), HIV transmission category (242, 2.3%), country of origin (218, 2.1%), CD4 count (775, 7.5%), VL (811, 7.8%), HCV coinfection (1081, 10.5%), and cause of death (47, 12.8% among death subjects). All missing values were imputed.

After imputation of missing values, 83.5% were men, 39.0% had compulsory or no education, 9.9% were IDUs, 58.8% were MSM, and 30.3% were heterosexuals. Spain was the country of origin of 69.3% of the patients, and median age at cohort entry was 35 (IQR 29–42) years. Median CD4 count was 386 cell/mm^3^ (IQR 200–586), median HIV VL was 46,504 copies/mL (IQR 11,800–151,000) and 1019 (9.9%) patients with AIDS at entry. Overall, 13.4% had a positive HCV test result at entry (Table 1).

### 3.2 Overall excess mortality

The overall eMR observed in the cohort was 0.82 deaths per 100 py of follow-up (95% CI 0.73, 0.93), that is, the death rate was 0.82 deaths per 100 py higher in CoRIS than in the Spanish population of the same age and sex for all causes of death. Overall eMRs by potential prognostic factors are shown in Table 1. The overall eMR was 1.91 per 100 py (95% CI 1.63, 2.24) during the first year of follow-up, decreasing to 0.5 per 100 py of follow-up (95% CI 0.41, 0.60) during the rest of the follow-up.

**Figure 1:**
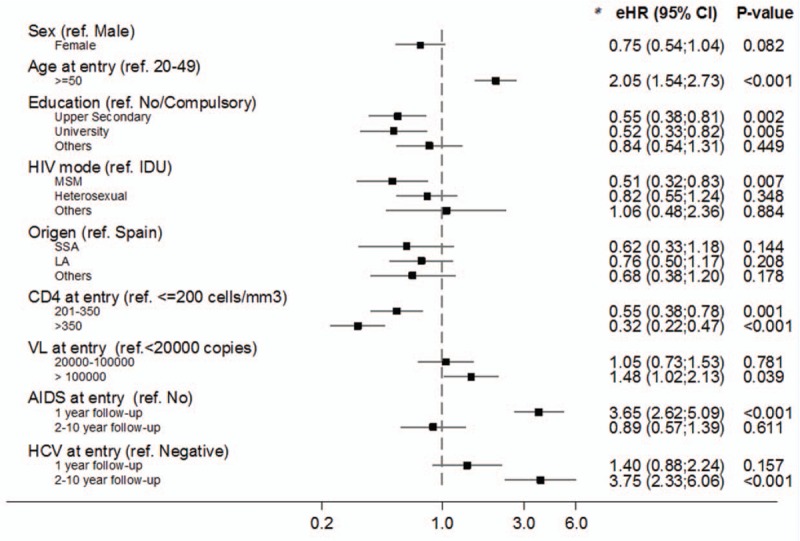
Adjusted excess hazard ratio (eHR) for the associations between potential risk factors and overall excess mortality rate. eHR and *P* values derived from a multivariable generalized linear model with Poisson error structure. *P* value derived from Wald test. LA = Latin America, SSA = sub-Saharan Africa.

In the multivariable model (Fig. 1), CD4 count and age at cohort enrolment were strong predictors for excess mortality adjusted for background mortality and all other risk predictors. Excess mortality was higher in those with VL at entry >100,000 compared with those with VL <20,000 (eHR 1.48, 95% CI 1.02, 2.13). Excess mortality was 0.55 (95% CI 0.38, 0.81) times lower in those with upper secondary and 0.52 (95% CI 0.33, 0.82) in those with university education than in the group of no or compulsory. Excess mortality was lower among MSM compared with IDUs (*P* = 0.007). There was borderline evidence that women had lower risk of excess mortality than men (eHR 0.75, 95% CI 0.54, 1.04). The short and medium/long-term effects of having AIDS (*P* < 0.001) and being HCV-positive (*P* < 0.001) at study entry on the excess mortality were significantly different; having AIDS at entry was a strong predictor of short-term excess mortality (eHR 3.65, 95% CI 2.62, 5.09), whereas this effect disappeared (eHR 0.89, 95% CI 0.57, 1.39) during the rest of follow-up. On the contrary, being HCV-positive at study entry predicted higher long-term excess mortality (eHR 3.75, 95% CI 2.33, 6.06), whereas there was no evidence of a statistically significant effect during the first year of follow-up (eHR 1.40, 95% CI 0.88, 2.24).

### 3.3 Cause-specific excess mortality

Non-AIDS-defining malignancies, liver, non-AIDS infections, and cardiovascular deaths distribution and eMRs by potential prognostic factors are shown in Table 2.

**Table 2:**
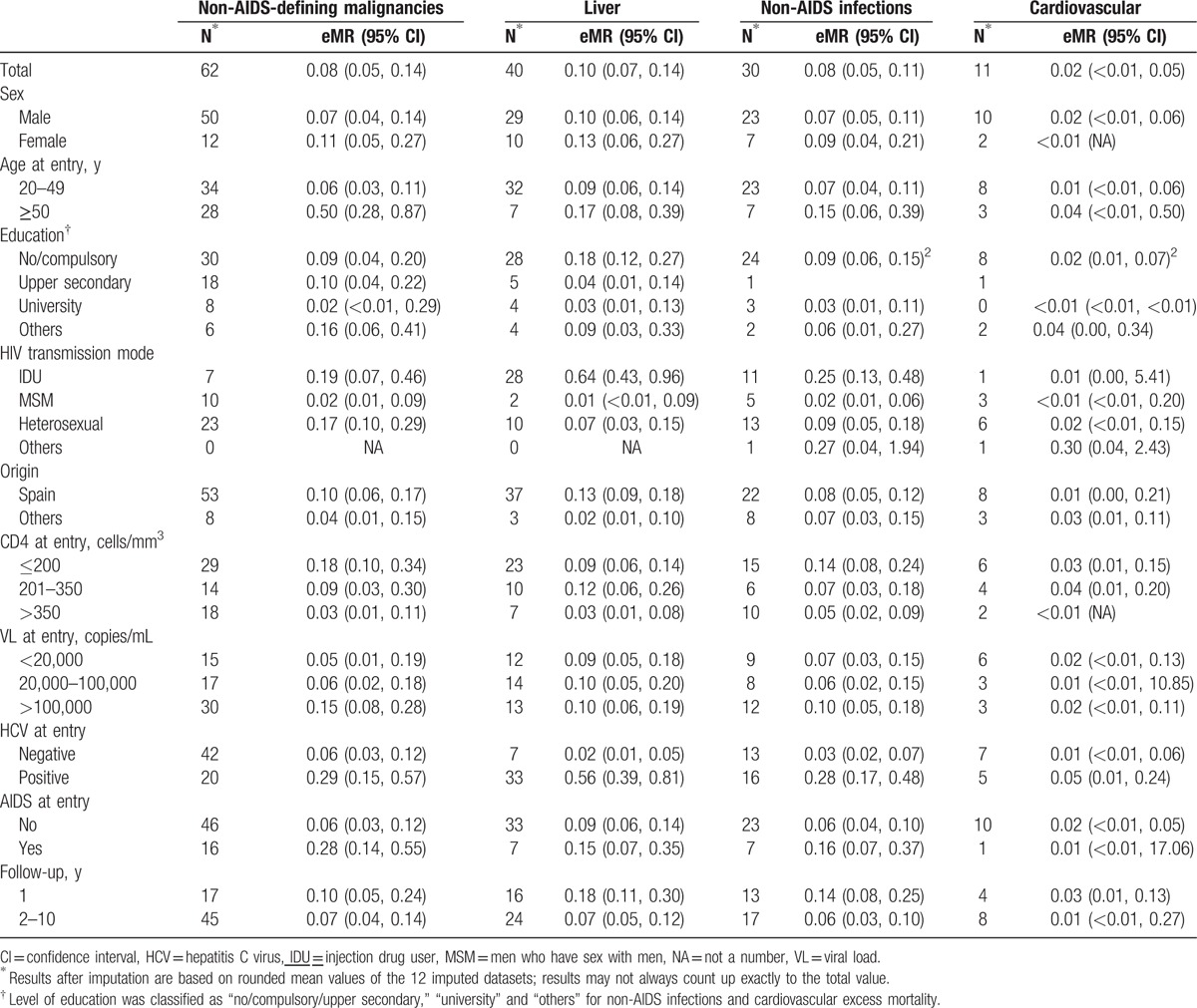
Non-AIDS-defining malignancies, liver, non-AIDS infections, cardiovascular-related deaths distribution and excess mortality rates (eMRs) per 100 person-years of follow-up.

The eMR observed from 2004 to 2014 in CoRIS was 0.08 deaths (95% CI 0.05, 0.14) for NADM, 0.10 deaths (95% CI 0.07, 0.14) for liver, 0.08 deaths (95% CI 0.05, 0.11) for non-AIDS infections, and 0.02 deaths (95% CI 0.00, 0.05) per 100 py of follow-up for cardiovascular causes.

**Figure 2:**
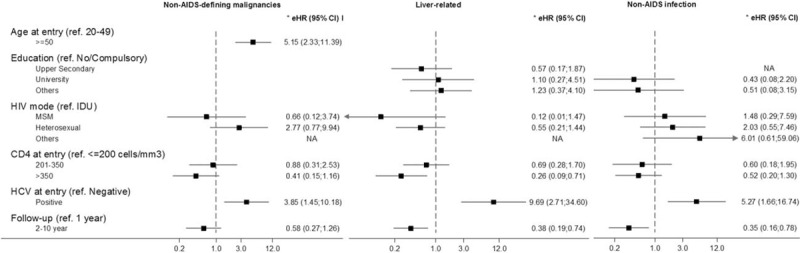
Adjusted cause-specific excess hazard ratio (eHR) for the associations between potential risk factors and cause-specific excess mortality rate. eHR derived from a multivariable generalized linear model with Poisson error structure. LA = Latin America, SSA = sub-Saharan Africa.

There was strong evidence that age and HCV coinfection at entry were independently associated with a higher NADM excess mortality. Patients aged 50 years old, or older at entry, had 5.15 times higher risk (95% CI 2.33, 11.39) compared with those aged below 50 years at entry, and the adjusted eHR for those HCV-coinfected subjects was 3.85 (95% CI 1.45, 10.18) (Fig. 2).

A CD4 count at entry greater than 350 cells/mm^3^ was associated with a 74% reduction of the liver excess mortality compared with CD4 counts lower than 200 cells/mm^3^ (eHR 0.26, 95% CI 0.09, 0.71). HCV-coinfected subjects had 9.69 times (95% CI 2.71, 34.60) higher risk of liver excess mortality than HCV-negative ones. Besides, there was strong evidence that long-term adjusted eHR was lower compared with short-term eHR (eHR 0.37, 95% CI 0.19, 0.73).

Having a positive HCV test at entry was also associated with higher non-AIDS infection-related excess mortality (eHR 5.27, 95% CI 1.66, 16.74), and there was strong evidence that long-term adjusted eHR was lower compared with short-term eHR (eHR 0.35, 95% CI 0.16, 0.78).

No significant predictor for the crude cardiovascular excess mortality was found, hence multivariable generalized linear model was not performed.

We failed to find any evidence that the effect of prognostic factors on cause-specific excess mortality had changed over time, although results might be interpreted with caution because numbers are small.

## 4 Discussion

The HIV-positive subjects in Spain have experienced, from 2004 to 2014, an overall excess mortality, and also an excess mortality for NADM, liver, non-AIDS infections, and cardiovascular disease compared with the general population of the same age and sex. These mortality excesses have happened largely at the expense of higher rates in the first year after cohort inclusion, and are attributable to late HIV presentation, which could be greatly attenuated by scaling up HIV testing and early diagnoses.

The excess mortality in HIV-positive patients has been reported both in Spain and elsewhere.^[15–17]^ The higher excess rates compared with CoRIS reported by Aldaz et al^[16]^ can be explained by their higher proportion of IDUs. The concerted action on seroconversion to AIDS and death in Europe (CASCADE) collaboration reported an excess mortality of 0.6 deaths per 100 py during 2004 to 2006.^[18]^ CASCADE collects data from seroconverters, hence the discrepancies with our results can be explained by different study populations. Not surprisingly, short-term mortality is less likely to happen in seroconverters because of the better clinical and immunological conditions at cohort entry; indeed CASCADE's overall excess mortality rate is similar to the 0.50 per 100 py found in CoRIS after excluding short-term mortality. Subjects with AIDS at cohort enrolment—subjects with late HIV presentation—had an increase of excess mortality during the first year of follow-up. The implications of a delayed HIV diagnosis have been previously documented.^[19]^ AIDS diagnosis has been related with poorer responses to cART.^[20]^

Previous reports in CoRIS had also noticed the importance of HCV/HIV coinfection on overall and cause-specific mortality in the Cohorts of the Spanish AIDS Research Network.^[15,21,22]^ Our finding about the differential effect of HCV coinfection on short and long-term excess mortality raises again the question about the role of HCV coinfection in mortality associated with HIV infection, even adjusting for background mortality. Excess mortality captures both direct and indirect effects of HIV infection on mortality. Long-term excess mortality in HCV-coinfected patients may be explained by the direct impact of HCV on HIV disease progression for which data are inconsistent. Some reports have found no evidence that HCV infection accelerates CD4 decline and AIDS progression, nor that it compromises CD4 count recovery after initiation of cART.^[23,24]^ Other studies have reported that HCV coinfection is associated with a higher risk of progression to AIDS^[25]^ and poorer CD4 recovery even after several years from cART initiation.^[26]^ Berenguer et al^[27]^ published that the eradication of HCV in HIV/HCV-coinfected patients is associated with decreases in HIV progression and lower risks of both liver and nonliver-related mortality. Long-term overall excess mortality in HCV-positive patients can be also explained by indirect mortality associated to higher exposure to drugs, alcohol, and tobacco, compared with the general population.^[9]^

Liver excess mortality associated with being HIV-positive can be mediated by the direct impact HIV has on the natural history of HCV.^[28]^ Besides, the toxicity of long-term antiviral treatment may contribute to liver damage and liver excess mortality.^[29]^ However, there is also indirect effect since hepatotoxic substances such as alcohol and illegal drugs are abused by HIV-positive subjects more often than the general population.^[30]^

We observed significantly higher NADM mortality among HIV-positive patients compared with the general population, which is consistent with previous reports.^[31]^ NADM excess mortality can be both directly and indirectly associated with HIV. Some studies have suggested that immunosuppression might be associated with moderated excesses of NADM^[32]^ and more rapid progression of some types of cancer.^[33]^ Besides, clear associations between immunosuppression and NADM with known viral etiology have been reported.^[33,34]^ NADM excess mortality can be also attributable to the previously alleged elevated frequency of cancer risk factors in HIV-positive patients such us smoking and alcohol abuse,^[30,35]^ and coinfection with oncogenic virus such as hepatitis D virus, HCV, HBV, and human papilloma virus.^[36]^

Hepatitis C virus coinfection and aging have been previously reported as risk factors for excess NADM rates.^[31,33]^ Patients older than 50 years have shown a poorer immunological response to cART^[37]^ which might also contribute to the higher NADM excess mortality observed in older patients.

Competing explanations for lower mortality rates in migrant populations fall into 2 broad categories. The first posits the self-selection of healthier migrants driven by labor market conditions in what is known as the “healthy immigrant effect.”^[38]^ A second hypothesis, known as “Salmon bias,” proposes that foreign-born persons return to their country of origin when they become severe ill.^[39]^ Therefore, our analysis should have used general population mortality rates matched by sex, age, and also region of origin, but unfortunately, cause-specific mortality rates by country of origin are not accessible in Spain.

There are some study limitations that merit discussion. As in other studies, some covariates (e.g., adherence to cART, alcohol and tobacco use, access/adherence to interferon/ribavirin, HCV-RNA determination) were not collected and consequently their impact on the results could not be analyzed. Another aspect that could be argued is the convenience of using different coding algorithms in the numerator and denominator of excess mortality estimations. However, it has been shown that revised CoDe classification is the best way to classify cause of death in HIV-positive cohort studies; it has been shown that applying ICD-10 system would underestimate liver mortality associated with being HIV-positive.^[12]^ Selection bias could have been introduced by the use of the general population as a proxy for background population as this population contains also HIV-related deaths. Nevertheless, as HIV-related mortality represents a small proportion of all-cause mortality in the general population of Spain, we consider correct it to use the general population mortality rates to calculate the mortality rates in a non-HIV-infected population. Misclassification of missing data due to imputation could also be discussed. However, sensitivity analysis restricted to complete cases did not reveal changes in the direction of the association between excess mortality and risk factors. Finally, the low number of deaths, when aiming to look at cause-specific mortality, may have introduced random error in some of our estimates and comparisons.

To conclude, our results have shown overall, NADM, liver, infection, and cardiovascular excess mortality associated with being HIV-positive despite improvements in HIV disease management. As far as we know, no previous analysis has demonstrated in this very clear way the role of having an AIDS diagnosis at entry in early excess mortality and HCV coinfection in long-term excess mortality. Our results have clear implications for health policy in that promoting earlier HIV diagnosis and linkage to care will likely decrease the excess of mortality detected in the first year. It is to be expected that the scale-up of the treatment with new direct acting antivirals for HCV will impact long-term mortality too. Finally, increased efforts promoting healthier lifestyles regarding diet, smoking, and exercise are urgently needed in the current context to aim for the healthy aging of the persons living with HIV.

## Supplementary Material

Supplemental Digital Content
