# Supplementary material for: Overall and cause-specific excess mortality in HIV-positive persons compared with the general population: Role of HCV coinfection
Source: Medicine (Baltimore). 2016 Sep 9;95(36):e4727. doi: 10.1097/MD.0000000000004727 (PMC5023891; doi:10.1097/MD.0000000000004727)
Supplement: Supplemental Digital Content [file medi-95-e4727-s001.doc]

**Supplemental Digital Content 1**


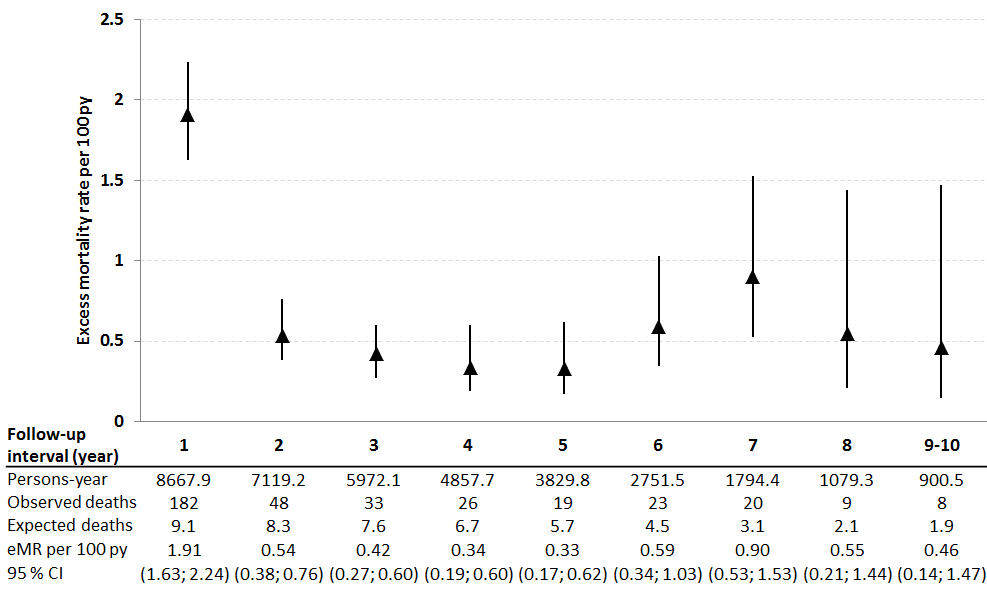
**Figure 1: Overall Excess Mortality Rate (eMR) by follow-up time interval**

**Supplemental Digital Content 2**

Table 1: Distribution of causes of death in CoRIS and in the general population from 2004 to 2014 before Multiple Imputation

|  | **General population** | **CoRIS** |
| --- | --- | --- |
| **Cause of Death** | **N (%)** | **N (%)** |
| AIDS-associated diseases | 11529 (0.30 %) | 154 (41.85 %) |
| Liver disease | 143085 (3.72 %) | 34 (9.24 %) |
| Non-AIDS-defining Malignancies | 1006780 (26.15 %) | 52 (14.13 %) |
| Non-AIDS Infections | 133787 (3.47 %) | 26 (7.07 %) |
| Cardiovascular disease | 1214711 (31.55 %) | 9 (2.45 %) |
| Diseases of the blood | 14174 (0.37 %) | 1 (0.27 %) |
| Pulmonary diseases | 332309 (8.63 %) | 9 (2.45 %) |
| Central nervous system diseases | 179137 (4.65 %) | 2 (0.54 %) |
| Drug abuse | 5875 (0.15 %) | 8 (2.17 %) |
| External causes | 113277 (2.94 %) | 7 (1.90 %) |
| Suicide | 34048 (0.88 %) | 8 (2.17 %) |
| Other diseases | 594126 (15.43 %) | 11 (2.99 %) |
| Il defined and unknown causes | 67874 (1.76 %) | 47 (12.77 %) |
| **TOTAL** | **3850712 (100.00 %)** | **368 (100.00 %)** |
